# Reduction or enhancement? Repetition effects on early brain potentials during visual word recognition are frequency dependent

**DOI:** 10.3389/fpsyg.2023.994903

**Published:** 2023-05-09

**Authors:** Chun-Hsien Hsu, Chia-Ying Lee

**Affiliations:** ^1^Institute of Cognitive Neuroscience, National Central University, Taoyuan City, Taiwan; ^2^Institute of Linguistics, Academia Sinica, Taipei City, Taiwan; ^3^Research Center for Mind, Brain, and Learning, National Chengchi University, Taipei City, Taiwan

**Keywords:** P200, orthographic neighborhood size, visual word recognition, Chinese characters, repetition enhancement, repetition reduction, alpha-band oscillation

## Abstract

Most studies on word repetition have demonstrated that repeated stimuli yield reductions in brain activity. Despite the well-known repetition reduction effect, some literature reports repetition enhancements in electroencephalogram (EEG) activities. However, although studies of object and face recognition have consistently demonstrated both repetition reduction and enhancement effects, the results of repetition enhancement effects were not consistent in studies of visual word recognition. Therefore, the present study aimed to further investigate the repetition effect on the P200, an early event-related potential (ERP) component that indexes the coactivation of lexical candidates during visual word recognition. To achieve a high signal-to-noise ratio, EEG signals were decomposed into various modes by using the Hilbert–Huang transform. Results demonstrated a repetition enhancement effect on P200 activity in alpha-band oscillation and that lexicality and orthographic neighborhood size would influence the magnitude of the repetition enhancement effect on P200. These findings suggest that alpha activity during visual word recognition might reflect the coactivation of orthographically similar words in the early stages of lexical processing. Meantime, there were repetition reduction effects on ERP activities in theta-delta band oscillation, which might index that the lateral inhibition between lexical candidates would be omitted in repetition.

## 1. Introduction

Object recognition substantially improves when stimuli are repeatedly presented (Tulving and Schacter, 1990; Schacter and Buckner, 1998). Studies focusing on brain activities have depicted the underlying mechanisms of the repetition priming effect. For example, event-related potential (ERP) studies of stimulus repetition and priming have consistently found that ERP responses to repeated pictures or words are reduced compared to non-repeated ones (Rugg, 1990; Schendan and Kutas, 2003). In visual word recognition studies, several have demonstrated the repetition reduction effect on N400 (Rugg, 1985, 1990; Van Petten et al., 1991; Swick, 1998; Misra and Holcomb, 2003; Laszlo et al., 2012), which is typically a negative wave over the central-posterior scalp and reflects semantic selection and integration (Kutas and Hillyard, 1980). The interactive activation model could explain the repetition reduction effect on N400 (McClelland and Rumelhart, 1981), which suggests a recurrent processing framework of word recognition and context effects in letter perception. Specifically, this model suggests that visual stimuli would generate upstream activation from inputs to sublexical-level representations to word-level representations. Meantime, there is also a flow of inhibition between representations at the same level. The lateral inhibitory connection between lexical candidates (such as orthographically similar words) would reduce the activation of each competitor, and only the activity of the target would accumulate over time. Therefore, while the same word/object is presented again, its activity level is already superior to other competitors, so stimuli repetition reveals faster response time and a reduction in N400.

As the majority on studies of visual word recognition endorse the repetition reduction effect on N400, there were studies that reported that repeated words revealed a more positive P200–the positive-going wave over the frontal site peaking around 200 ms after stimuli onsets–than non-repeated words (Van Petten et al., 1991; Curran and Dien, 2003; Misra and Holcomb, 2003; Pesciarelli et al., 2007). A question that remains unanswered is what process would be involved in P200. A potential explanation might be that the amplitude of P200 is inversely correlated to the degree of coactivation among lexical candidates. This notion is consistent with Bles et al.’s (2007) result. In their study, participants saw strings of green letters presented in successive gates–stimuli were presented letter by letter. The letters on the screen never formed actual words, and the task was to make a button response when the new letter was in red. In their results, P200 activity was found to vary as a function of the number of lexical candidates calculated by counting words that started with the first, the first two, or all three letters. Specifically, P200 increased as the size of the cohort of lexical candidates decreased.

Indeed, studies have provided interesting insights onto the relationship between the degree of coactivation of lexical candidates and the amplitude of P200. For example, studies of the reading of English words and Spanish words have reported effects of orthographic neighborhood size (Holcomb et al., 2002) and syllable frequency (Barber et al., 2004; Carreiras et al., 2005), respectively, on P200. That is, P200 responses to words with few orthographic neighbors and low syllable frequency were stronger than those with many orthographic neighbors and high syllable frequency. In the studies of alphabetic languages, orthographic neighborhood size was computed as the total number of words that could be formed by replacing one letter of a target item. Therefore, it appears that activation of a few lexical candidates would be associated with stronger P200. If this interpretation is correct, one might interpret that repeated words are somehow similar to that in the reading of words related to a small-sized cohort. Subsequently, the role of P200 activity during word repetition would become what is the mechanism involved in giving this effect?

Studies of episodic retrieval have argued that words sharing orthographic representations with many other words will produce more item noise than more unique words. This argument was supported by the effect of orthographic neighborhood size on the performance in a memory recognition task, the so-called orthographic distinctiveness effect (Glanc and Greene, 2007; Cortese et al., 2010). Therefore, it seems appropriate to speculate that repetition enhancement effects on P200 might be linked to the degree of synchronous activity associated with the amount of coactivation of lexical candidates. Interestingly, according to the neural synchrony hypothesis (Ghuman et al., 2008; Gotts et al., 2012, 2021), behavioral facilitation in repetition priming is a consequence of an increase in synchronous activity, despite a decrease in the overall firing rate of neurons. Specifically, neurons would activate in a more synchronous and temporally coordinated manner following stimulus repetition, so the changes in amplitudes might be due to the high coherences in neural oscillation. The neural synchrony hypothesis further predicts that stimuli repetition would also enhance brain activity because of increased phase consistency. Two findings seem to align with the neural synchrony hypothesis. First, studies using functional magnetic resonance imaging (fMRI) have demonstrated the repetition enhancement effect on brain activities (Henson et al., 2000; Turk-Browne et al., 2007), and the long-range synchronization induced by stimuli repetition. Second, EEG and magnetoencephalogram (MEG) studies have shown that brain oscillation at alpha bands contributed to repetition enhancements in objects and face recognition (Gilbert et al., 2010; Engell and McCarthy, 2014; Sauer et al., 2020).

After describing relevant studies that reported the repetition enhancement effect, the present study aimed to elucidate the extent to which the repetition enhancement effect on P200 would be modulated by lexicality and phonetic radical combinability (Hsu et al., 2009). The language utilized in the present study was Mandarin Chinese. Regarding its orthographic system, Chinese characters were initially designed to resemble the physical objects they represented, either literally or metaphorically. However, some concepts and meanings could not be conveyed by pictorial resemblance alone, so phonograms were created during the Shang dynasty (1,600–1,046 BC) by compounding a semantic radical and a phonetic radical. The semantic radical provides a hint for the character’s meaning, while the phonetic radical provides information for its pronunciation. For example, the character 採 (to pick, pronounced as/cai3/) has a semantic radical on the left and a phonetic radical on the right. Its phonetic radical is the same as the phonetic radical of 菜 (vegetables/cai4/), 睬 (to take notice of/cai3/), 彩 (colors/cai3/), and 綵 (colored/cai3/). The semantic radical 扌 is semantically related to the concepts of a hand or an action because it is abbreviated from the word 手 (a hand; to hold/shou3/). According to Myers (2019), over 80% of traditional Chinese characters are phonograms. Among these phonograms, 63% have a semantic radical on the left and a phonetic radical on the right (e.g., 採), and 6% have an opposite layout (e.g., 彩). Phonetic radical combinability is similar to the concept of orthographic neighborhood size and refers to the number of Chinese characters in which a phonetic radical appears, regardless of their pronunciations (Feldman and Siok, 1997).

Some studies have raised controversies regarding the repetition enhancement effect on P200. For example, in the study of Almeida and Poeppel (2013), participants performed lexical decisions to real words and pseudowords, and pseudowords were all pronounceable and phonotactically legal strings. All of their stimuli were high orthographic neighborhood items, and the result demonstrated the interaction between lexicality (word vs. pseudoword) and stimuli repetition. That is, repetition of words, but not pseudowords, evoked stronger activity on the bilateral frontal scalp in the 150–250 ms window. They referred the P200 activity to the notion that it might reflect early access to long-term memory. Therefore, the reading of pseudowords did not possess an entry in the mental lexicon and did not show the repetition effect on this component. However, some studies did not demonstrate the repetition effect on P200 in the reading of real words (Swick, 1998; Laszlo et al., 2012). The definition of lexicality in these studies was different from that in Almeida and Poeppel (2013). In Swick (1998), participants performed lexical decision to real words and non-words created by rearranging the sequence of letters in a word. This study demonstrated the repetition reduction effect on N400 and did not show the repetition effect on early ERP components. In Laszlo et al. (2012), there were four stimulus types, including real words, pseudowords, meaningless illegal strings, and familiar but orthographically illegal acronyms. In the meaningfulness judgment, Laszlo et al.’s (2012) result demonstrated a robust repetition reduction on N400 across all stimuli conditions. Interestingly, this study showed the repetition enhancement effect on P200 activity to illegal strings, and the rest of the condition did not show the repetition effect on P200.

Note that previous studies on visual word recognition have demonstrated strong effects of lexicality on the repetition enhancement effect on P200. However, the contribution of neighborhood density on repetition priming is thought to be important and has not been considered in previous studies. In summary, the presented study tested whether P200 activity to repeated real and pseudo characters would show the repetition enhancement effect while considering phonetic radical combinability. Assuming that repetition enhancement indexes synchronous and temporally coordinated activity to stimulus repetition (Gotts et al., 2021), one would predict repetition enhancement effects on P200 regardless of lexicality and the size of orthographic neighbors. We also evaluated whether stimulus repetition would influence oscillation responses, as previous studies have demonstrated semantic priming (Brennan et al., 2014), repetition priming (Sauer et al., 2020) and lexicality (Strauss et al., 2014) effects on alpha activity during word recognition. On the other hand, studies have pointed out that the noise among orthographically similar items would diminish the repetition effect (Glanc and Greene, 2007; Cortese et al., 2010). This notion would further predict an interaction between orthographic neighborhood size and stimulus repetition.

## 2. Materials and methods

### 2.1. Participants

Twenty-six right-handed native Chinese speakers were recruited to participate in a go/no-go semantic categorization task. All participants were college students with normal or corrected to normal vision. The Human Subject Research Ethics Committee/IRB of Academia Sinica, Taiwan, approved the current study.

### 2.2. Materials

In the go/no-go task, fifty six animal names in Chinese characters were applied in the go trials. In the no-go trials, we used a 2 × 2 × 2 design with repetition (new stimuli and repeated stimuli), phonetic radical combinability (large and small), and lexicality (real characters and pseudo characters) as within-subject factors. Lexical and sublexical properties were based on the Sinica Corpus 4.0 (Huang and Chen, 1998). The corpus is based on more than five million words (approximately 10 million characters) from textbooks, newspapers, literature, popular fiction and non-fiction, and transcripts. The index for phonetic radical combinability and the control factors–including the number of strokes, character frequency, and semantic radical combinability and phonological consistency–was calculated based on 3,697 phonograms. Phonological consistency is defined by the ratio of the number of characters with the same phonetic radical and the same pronunciation to the number of all characters with the same phonetic radical (Chang et al., 2016). Phonetic radical combinability was defined as the number of phonograms that share a phonetic radical. Across 3,697 phonograms, phonetic radical combinability ranged between 1 and 20, and the median was 6. Therefore, a large phonetic radical combinability condition included stimuli with a phonetic radical combinability between 7 and 20. For a small phonetic radical combinability condition, stimuli with a phonetic radical combinability between 2 and 5 were included (Table 1). The critical stimuli were presented twice in succession, including thirty characters with large phonetic radical combinability, thirty characters with small phonetic radical combinability, thirty pseudo-characters (created by combining radicals, and these combinations never existed in any characters) with large phonetic radical combinability, and thirty pseudo-characters with small phonetic radical combinability. To avoid participants’ bias on immediately repeated stimuli, there were additional fillers which included characters and pseudo-characters. Half of the fillers were only shown once, and the rest were repeated after a few trials. The stroke number and semantic radical combinability were matched across each condition and filler. For real characters, phonological consistency and character frequency were matched across each condition and filler.

**TABLE 1:** Means of parameters for target stimuli.

	Real characters		
	**Large phonetic radical combinability** **(*N* = 30)**	**Small phonetic radical combinability** **(*N* = 30)**	***F*-values in one way ANOVA**
Phonetic radical combinability	11.6	3.3	124.47 (*p* < 0.001)
Semantic radical combinability	98.8	98.33	0.001 (*p* > 0.1)
Character frequency	33.43	41.73	1.501 (*p* > 0.1)
Phonological consistency	0.42	0.44	0.272 (*p* > 0.1)
Number of strokes	12.9	12.2	1.501 (*p* > 0.1)
	**Pseudo characters**		
	**Large phonetic radical combinability** **(*N* = 30)**	**Small phonetic radical combinability** **(*N* = 30)**	***F*-values in one way ANOVA**
Phonetic radical combinability	7.97	4.13	70.08 (*p* < 0.001)
Semantic radical combinability	87.23	81.50	0.418 (*p* > 0.1)
Number of strokes	11.57	12.2	0.651 (*p* > 0.1)
	**Lexicality**		
	**Real characters** **(*N* = 60)**	**Pseudo characters** **(*N* = 60)**	***F*-values in one way ANOVA**
Phonetic radical combinability	12.55	11.83	1.563 (*p* > 0.1)
Semantic radical combinability	98.57	84.37	3.618 (*p* > 0.05)
Number of strokes	12.55	11.83	1.563 (*p* > 0.1)

### 2.3. Procedures

A trial began with two vertical lines–one upper and the other lower to the center of the screen–which were simultaneously presented for 500°ms. Then a stimulus was presented at the center between the two lines for another 150 ms. The two lines and the character were then replaced with a cross at the center of the screen for 850 ms. A blank screen was presented for another 250 ms after the cross, and then a capital letter “B” was presented at the center of the screen for 1.5 s as a signal for the participants to blink quickly if necessary before the next trial. The inter-trial interval was varied from 500 to 1,000 ms. Participants were asked to maintain their fixation at the midpoint between the two lines and press a button as quickly as possible if the presented stimulus represented an animal name. This design was to ensure that participants were identifying the characters’ meaning and to avoid any ERP responses from motor processes, such as lateralized readiness potentials, which would distort ERP waveforms.

### 2.4. EEG recording

The electroencephalogram (EEG) was recorded from 64 Ag/AgCl electrodes (QuickCap, Neuromedical Supplies, Sterling, USA) with a common vertex reference located between Cz and CPz. For further analysis, the data were re-referenced offline to the average of the right and left mastoids. Vertical eye movements (VEOG) were recorded by a pair of electrodes placed on the left eye’s supra- and infra-orbital ridges. Horizontal eye movements (HEOG) were recorded by a pair of electrodes placed lateral to the outer canthus of the right and left eyes. A ground electrode was placed on the forehead anterior to Fz. Electrode impedance was kept below 5 KΩ. The EEG signal was continuously recorded and digitized at a rate of 1,000 Hz. The signal was amplified by SynAmps2^®^ (Neuroscan, Inc., El Paso, TX, USA) amplifiers with the low-pass filter at DC–100 Hz for offline analysis. For the offline analysis, the continuous wave was epoched with a 100 ms pre-stimulus and 800 ms post-stimulus interval. The pre-stimulus interval was used for baseline correction. Segments of the EEG signal with voltage variations larger than 100 μV were excluded from subsequent analyses.

### 2.5. EEG preprocessing

#### 2.5.1. EMD-based measures of ERPs

Instead of conventional ERP analyses, we applied the Hilbert–Huang transform (HHT) (Huang et al., 1998). Lo et al. (2009) pointed out that human brain is a complex system generating non-stationary and non-linear signals. Therefore, assumption of stationarity and linearity are generally not appropriate for EEG data. This is because wavelet analyses and Fourier based methods use a prior function to estimate brain activities within the desired frequency range (e.g., the sinusoid function and the Morlet function), and a fixed prior function cannot represent signals generated from non-linear and non-stationary systems. In line with this concern, Cong et al.’s (2009) and Williams et al.’s (2011) results demonstrated that HHT approach provided better estimation of amplitude and latency than ERPs that are extracted by wavelet analyses and band-pass filtering. Park et al. (2013) used the short time furrier transform, wavelet transform, and HHT to calculate time-frequency spectra to classify EEG signals recorded during motor imagery and motor execution. Their result demonstrated that HHT gave the best classification performance. Hsu et al. (2016) demonstrated that EEG responses measured by HHT had a considerably higher statistical power and required fewer trials for each condition than the conventional ERP did for investigating the mismatch negativity effect.

**FIGURE 1:**
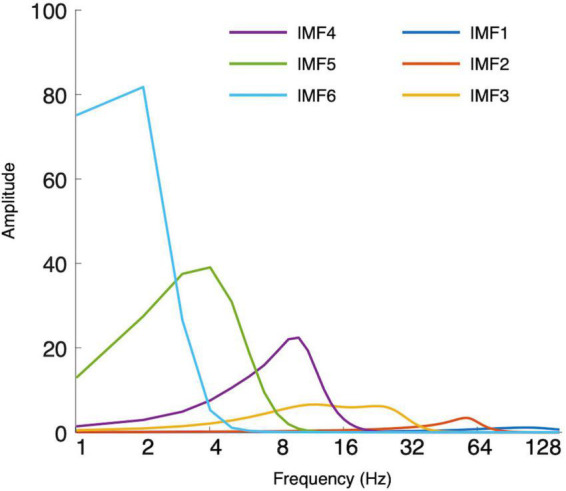
Hilbert–Huang transform (HHT) marginal spectra of each intrinsic mode function (IMF).

**FIGURE 2:**
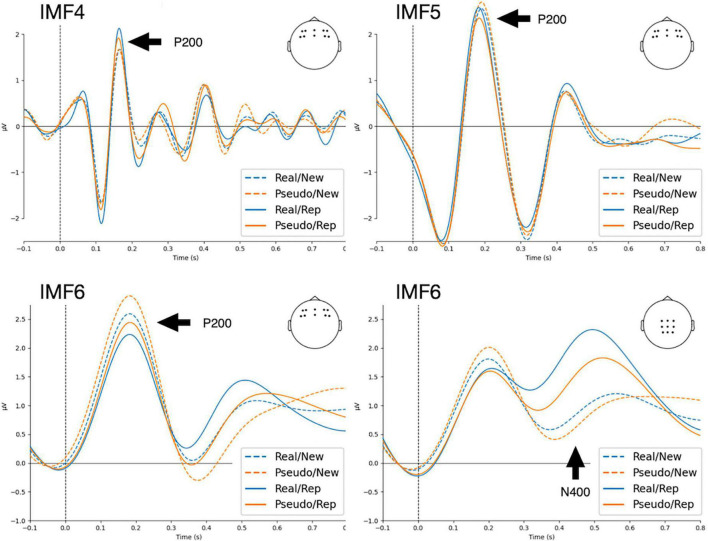
Results of event-related modes (ERMs) averaged across waveforms in frontal (F5, F6, F3, F4, FC5, FC6, FC3, FC4, FZ, and FCZ) and posterior (CZ, C1, C2, CPZ, CP1, CP2, PZ, P1, and P2) regions. Real, real characters; Pseudo, pseudo characters; New, new stimuli; Rep., repeated stimuli.

Each EEG segment was decomposed into seven intrinsic mode functions (IMFs) using the masked empirical mode decomposition (EMD) (Quinn et al., 2021). Similar to conventional ERP procedures, averaging IMFs across trials gives event-related modes (ERM) (Al-Subari et al., 2015a,b; Chen et al., 2016; Chuang et al., 2019). The residual trend was IMF7, and IMF1 to IMF6 could be considered to estimate the ERMs. Figure 1 shows the marginal Hilbert spectra of IMFs averaged across participants and channels. The median frequencies of IMF1, IMF2, IMF3, IMF4, IMF5, and IMF6 were 96, 52, 19, 9, 4, and 2 Hz, respectively. Through visual investigation (Figure 2), IMF4, IMF5, and IMF6 showed P200 components in frontal electrodes. Currently, it remains unknown which frequency bands would be correlated with P200 components. Therefore, subsequent analyses of P200 were conducted using mean amplitudes in IMF4, IMF5, and IMF6, such that local mean amplitudes were calculated by averaging the amplitude in a 20 ms window centered at the positive peak within the time window from 100 to 300 ms in ten electrodes of interest (F5, F6, F3, F4, FC5, FC6, FC3, FC4, FZ, and FCZ). For N400, previous studies using ERM measures have identified that N400 is represented in the delta oscillations (Chen et al., 2016; Tzeng et al., 2017, 2018). Accordingly, N400 was estimated using the mean amplitudes of IMF6 by averaging the amplitude from 400 to 600 ms in nine electrodes of interest (CZ, C1, C2, CPZ, CP1, CP2, PZ, P1, and P2).

#### 2.5.2. Trial-to-trial analyses of time-frequency responses

On each trial, the IMFs were analyzed by a direct quadrature transform (Huang et al., 2009) to obtain the instantaneous phase, instantaneous frequency and instantaneous amplitude. Then we estimated the single trial-based time-frequency spectra and phase synchronization indexed by intertrial coherence (ITC) (Lachaux et al., 1999; Zhang et al., 2014). We adopted the HHT methods for analyze time-frequency responses because it can provide better resolution in both the time and frequency domains. In brief, EMD is a data-driven, adaptive method that decomposes a signal into a finite number of IMFs determined using an iterative sifting process that separates the signal into high and low frequency components. This decomposition method is adaptive and can automatically adjust to the signal’s frequency content. Once the signal is decomposed into IMFs, the instantaneous phase of each IMF can be calculated using the direct quadrature transform. The instantaneous frequency can then be obtained by calculating the time derivative of the instantaneous phase. This approach allows for better frequency resolution as it estimates the frequency content of the signal at each time point, rather than using methods such as the convolution integral method used in Fourier transform and wavelet transform. Therefore, HHT is useful in the analysis of non-linear and non-stationary signals.

#### 2.5.3. Statistical analyses of mean amplitudes

Mean amplitudes of P200 and N400 were analyzed with the linear mixed-effects model (Bates et al., 2015) with two random factors (random intercepts for participants and electrodes). The use of mixed-effects models with electrodes as a crossed random effect could omit strong effects mainly influenced by one electrode instead of all electrodes of interest. This approach has been applied to scalp EEG (Kiyama et al., 2018; Cheimariou et al., 2019; Farshchi et al., 2020; Jaramillo et al., 2020; Mohan et al., 2020, 2022; Naumann et al., 2022) and intracranial EEG (Brzezicka et al., 2019; Hnazaee et al., 2020; Yin et al., 2020) analyses. Repetition (repeated minus new), lexicality (real characters minus pseudo characters), phonetic radical combinability (small minus large), and their interaction were fixed factors. All data were analyzed in R (Version 3.5.2; R Core Team, 2018) and RStudio (Version 1.1.463; R Studio Team, 2018). The linear mixed-effects model was run using the “lmer” function as implemented in the lme4 package for R (Version 1.1–21; Bates et al., 2015). Reported *p*-values were calculated based on Satterthwaite’s method as implemented in the lmerTest package in R (Version 3.1–3; Kuznetsova et al., 2017). *Post hoc* comparisons were carried out using the “glht” function (the multcomp package, Version 1.4–15; Hothorn et al., 2008) with Bonferroni correction.

#### 2.5.4. Statistical analyses of time-frequency responses

For the time-frequency measures, we investigated repetition effects on the trial-averaged power and phase coherences in electrodes of interest, including ten electrodes on the frontal scalp and nine on the central-to-parietal scalp. Cluster-based permutation tests (Maris and Oostenveld, 2007) were applied to identify adjacent clusters of time-frequency points that showed significant differences between new and repeated stimuli. Using *t*-tests, we first computed the group-level effects of presentation times on the trial-averaged spectra/coherence at electrodes of interest. The t-statistic for time-frequency points exceeding a threshold of *p* < 0.05 (cluster alpha) was summed. Then, we compared the maximum of the summed t-statistic in the observed data with a random partition formed by permuting the experiment condition labels 10,000 times. Clusters whose t-statistic exceeded 95% (*p* < 0.05) of the random partition were considered significant.

## 3. Results

### 3.1. Mean amplitudes of P200 in IMF4, IMF5, and IMF6

**FIGURE 3:**
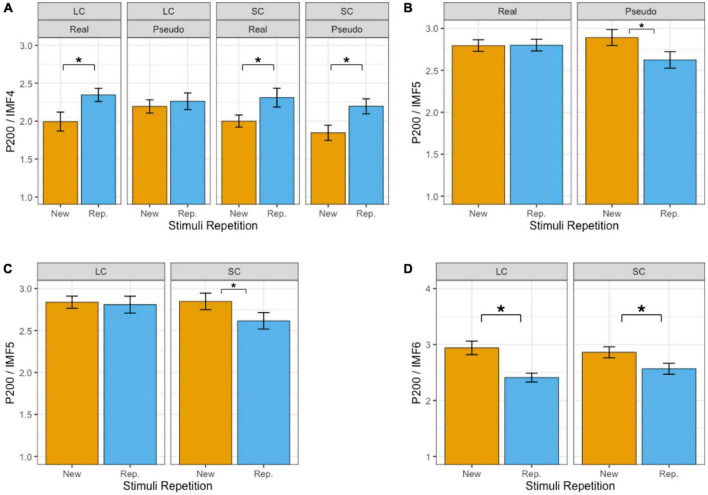
Bar plots of mean amplitudes of P200 in IMF4 **(A)**, IMF5 **(B,C)**, and IMF6 **(D)**. Error bars reflect by-subject standard errors. *Indicates *p* < 0.05. Real, real characters; Pseudo, pseudo characters; New, new stimuli; Rep., repeated stimuli; LC, large phonetic radical combinability; SC, small phonetic radical combinability.

Table 2 summarizes the results of fixed effects on P200. In IMF4, there was a significant effect of repetition enhancements on P200 (beta = 0.269, SE = 0.032, *p* < 0.001), i.e., enhancements of P200 activities during repetition (2.28 μV) compared with the first presentation (2.01°μV). The main effect of phonetic radical combinability was significant (beta = −0.110, SE = 0.032, *p* < 0.001), which showed that P200 was larger in response to stimuli with large phonetic radical combinability (2.19°μV) than that with small phonetic radical combinability (2.09°μV). The main effect of lexicality was not significant (beta = −0.038, SE = 0.032, *p* > 0.1). There were significant two-way interactions between lexicality and phonetic radical combinability (beta = −0.191, SE = 0.064, *p* < 0.01), which showed that the simple main effect of phonetic radical combinability was significant in the reading of pseudo-characters (*p* < 0.001) but not of real characters (*p* > 0.1). Most importantly, there was a significant three-way interaction between repetition, lexicality, and phonetic radical combinability (beta = 0.324, SE = 0.128, *p* < 0.05). Figure 3A shows bar plots for the three-way interaction between repetition, phonetic radical combinability, and lexicality. *Post hoc* analyses of repetition effects indicated that small radical combinability conditions revealed repetition enhancements regardless of lexicality (*p* < 0.001). In large radical combinability conditions, real characters revealed a repetition enhancement effect (beta = 0.351, SE = 0.064, *p* < 0.001), and pseudo-characters did not show the repetition effect (beta = 0.068, SE = 0.064, *p* > 0.1).

**TABLE 2:** Summaries of fixed effects of linear mixed-effects model (LMM) analyses for P200.

	β	S.E.	*t*-value	*p*-value
**P200/IMF4**				
Lexicality	–0.038	0.032	–1.186	n.s.
PhoneticComb	–0.110	0.032	–3.438	<0.001
Repetition	0.269	0.032	8.371	<0.001
Lexicality:PhoneticComb	–0.191	0.064	–2.973	<0.01
Lexicality:Repetition	–0.121	0.064	–1.889	n.s.
PhoneticComb:Repetition	0.119	0.064	1.85	n.s.
Lexicality:PhoneticComb:Repetition	0.324	0.128	2.518	<0.05
**P200/IMF5**				
Lexicality	–0.04	0.047	–0.839	n.s.
PhoneticComb	–0.090	0.047	–1.927	n.s.
Repetition	–0.130	0.047	–2.737	<0.01
Lexicality:PhoneticComb	–0.032	0.095	–0.337	n.s.
Lexicality:Repetition	–0.272	0.095	–2.851	<0.01
PhoneticComb:Repetition	–0.202	0.095	–2.113	<0.05
Lexicality:PhoneticComb:Repetition	–0.354	0.19	–1.859	n.s.
**P200/IMF6**				
Lexicality	0.255	0.057	4.472	<0.001
PhoneticComb	0.039	0.057	0.687	n.s.
Repetition	–0.412	0.057	–7.212	<0.001
Lexicality:PhoneticComb	–0.054	0.114	–0.473	n.s.
Lexicality:Repetition	–0.111	0.114	–0.976	n.s.
PhoneticComb:Repetition	0.234	0.114	2.055	<0.05
Lexicality:PhoneticComb:Repetition	–0.283	0.228	–1.239	n.s.

In IMF5, there was a significant effect of repetition reduction on P200 (beta = −0.130, SE = 0.047, *p* < 0.01), i.e., reduction of P200 activities during repetition (2.71°μV) compared with the first presentation (2.84°μV). Main effects of lexicality (beta = −0.04, SE = 0.047, *p* > 0.1) and phonetic radical combinability (beta = −0.09, SE = 0.047, *p* > 0.05) were not significant. There were significant two-way interactions between repetition and lexicality (beta = −0.272, SE = 0.095, *p* < 0.01) and between repetition and combinability (beta = −0.202, SE = 0.095, *p* < 0.05). The three-way interaction was not significant (beta = −0.354, SE = 0.190, *p* > 0.05). Figures 3B, C show the bar plots for two-way interactions. *Post hoc* analyses of repetition effects showed that the reading of pseudo-characters revealed repetition reduction (beta = −0.266, SE = 0.067, *p* < 0.001), and the reading of stimuli with small radical combinability revealed repetition reduction (beta = −0.231, SE = 0.067, *p* < 0.01).

In IMF6, there was a significant effect of repetition reduction on P200 (repetition: 2.49°μV; new stimuli: 2.90°μV; beta = −0.412, SE = 0.057, *p* < 0.001). The main effect of lexicality (beta = 0.255, SE = 0.057, *p* < 0.001) was significant (pseudo-characters: 2.82°μV; real characters: 2.57°μV). The main effect of phonetic radical combinability was not significant (beta = 0.039, SE = 0.057, *p* > 0.1). There was a significant two-way interactions between phonetic radical combinability and repetition (beta = 0.234, SE = 0.114, *p* < 0.05). The three-way interaction was not significant (beta = 0.713, SE = 0.258, *p* < 0.01). Figure 3D shows the bar plots for two-way interaction between repetition and phonetic radical combinability. *Post hoc* analyses of repetition effects indicated that both large (beta = −0.529, SE = 0.080, *p* < 0.001) and small (beta = −0.294, SE = 0.080, *p* < 0.001) radical combinability conditions revealed repetition reductions, and the former condition revealed the largest repetition effect.

### 3.2. Mean amplitudes of N400 in IMF6

**FIGURE 4:**
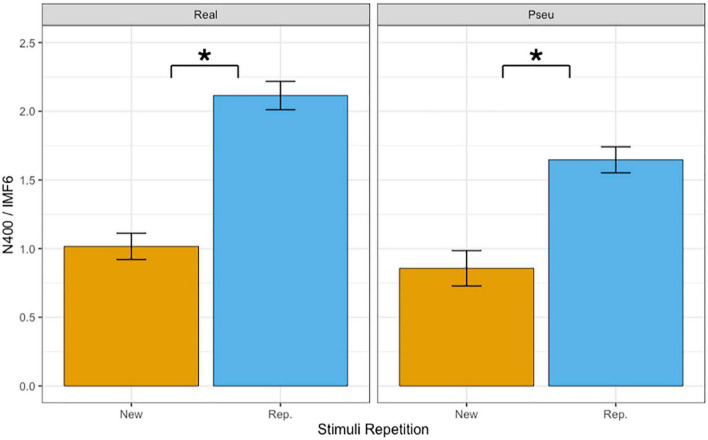
Bar plots of mean amplitudes of N400 in IMF6. Error bars reflect by-subject standard errors. *Indicates *p* < 0.05.

Table 3 summarizes the results of fixed effects on N400. The main effects of lexicality (beta = −0.313, SE = 0.057, *p* < 0.001) and repetition (beta = 0.944, SE = 0.057, *p* < 0.001) were significant. The results indicated that the reading of pseudo-characters revealed a stronger N400 (1.25°μV) than of real characters (1.56°μV) and that the N400 was reduced during the repetition (1.88 mV) as compared with the first presentation (0.94°μV). The main effect of phonetic radical combinability were not significant (beta = 0.001, SE = 0.057, *p* > 0.1). Finally, there was a significant two-way interaction between repetition and lexicality (beta = −0.308, SE = 0.115, *p* < 0.01). Figure 4 shows the bar plots for the interaction between repetition and lexicality. *Post hoc* analyses of repetition effects indicated that both real characters (beta = 1.098, SE = 0.081, *p* < 0.001) and pseudo-characters (beta = 0.789, SE = 0.081, *p* < 0.001) showed repetition reduction effect on N400, and the former condition revealed the largest repetition effect.

**TABLE 3:** Summaries of fixed effects of linear mixed-effects model (LMM) analyses for N400.

	β	S.E.	*t*-value	*p*-value
**N400/IMF6**				
Lexicality	−0.313	0.057	−5.465	<0.001
PhoneticComb	0.001	0.057	0.032	n.s.
Repetition	0.944	0.057	16.440	<0.001
Lexicality:PhoneticComb	0.044	0.115	0.389	n.s.
Lexicality:Repetition	−0.308	0.115	−2.685	<0.01
PhoneticComb:Repetition	0.043	0.115	0.378	n.s.
Lexicality:PhoneticComb:Repetition	−0.367	0.230	−1.601	n.s.

### 3.3. Time-frequency spectra and phase coherence

**FIGURE 5:**
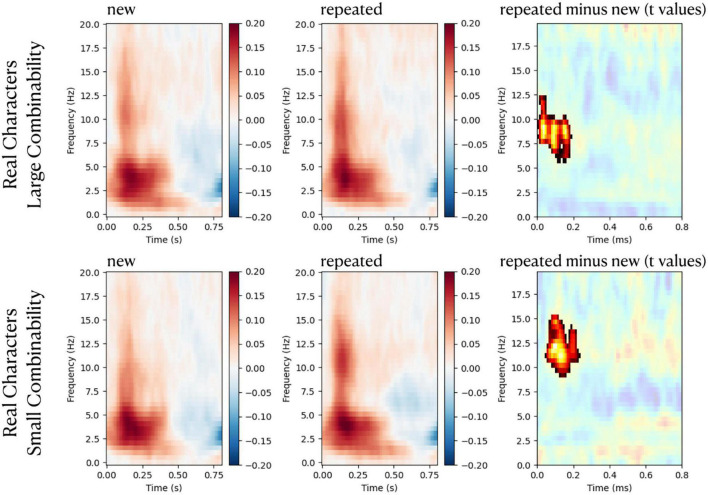
Figures in the left and the middle columns are plots of time-frequency intertrial coherence (ITC) responses to repeated and new stimuli, respectively. ITCs were normalized (in relative units) to baseline values (from –100 to 0 ms). Plots in the right column show *t*-values of the contrast between repeated stimuli and new stimuli, and non-significant time-frequency samples are masked in white. The red color indicates time-frequency samples with significant repetition enhancement effects on ITC (*p* < 0.05, cluster corrected).

There were no significant differences in power spectra between repeated stimuli and new stimuli. Regarding ITC in the frontal scalp, significant increases in phase synchronization were observed for repeated real-characters for frequencies between ∼6–15 Hz during the time period of ∼20–200 ms. Significant results of cluster-based analyses of repetition effects (repeated minus new) on ITC are shown in Figure 5. The plots for the pseudo-character conditions are not displayed in Figure 5 as they did not yield any significant results.

## 4. Discussion

As reviewed earlier, most studies of visual object recognition have demonstrated both repetition enhancement and reduction. However, the repetition enhancement effects on EEG responses to language stimuli have not yet been well characterized. The present study was designed to examine the extent to which lexicality and the amount of coactivation of lexical candidates (i.e., orthographic neighbors) influence repetition enhancement/reduction. In addition, previous studies have not shown which aspect of EEG oscillation contributes to the repetition enhancement effect in visual word recognition. To complement the neural signature of the repetition enhancement effect on EEG activity, the present study also elucidated the extent to which repetition effects on P200 might be due to increased activity or phase coherence. According to the neural synchrony hypothesis, stimuli repetition would be accompanied by an increase in phase synchrony between trials at alpha oscillation. The theoretical implications of the present findings will be further explained below.

The present study revealed increases in P200 associated with repetition for real characters. This result is consistent with previous reports showing a repetition enhancement effect on P200 activity for real words (Van Petten et al., 1991; Curran and Dien, 2003; Misra and Holcomb, 2003; Almeida and Poeppel, 2013). Furthermore, these repetition enhancement effects were found in IMF4, which manifested itself as alpha-band activity. Time-frequency analyses also highlighted the increases in alpha band ITC associated with repetition for real words. These results can be linked to the increase in the activation of the target words, as the study by Brennan et al. (2014) has demonstrated that fluctuations of alpha-band activity are associated with early stages of lexical access.

On the other hand, the reading of pseudo-characters only showed the repetition enhancement effect in the small phonetic radical combinability condition. Interestingly, this finding is consistent with the report by Laszlo et al.’s (2012) where non-words with few orthographic neighbors showed a repetition enhancement effect on P200. The present finding is also consistent with the report by Almeida and Poeppel’s (2013) where pseudowords with many orthographic neighbors did not show a repetition effect on MEG activity in the time windows corresponding to P200. We speculate that this interaction might be associated with the orthographic distinctiveness effect (Cortese et al., 2010). Interference from the noise of orthographic patterns shared with neighbors may hinder the episodic recollection of stimuli with many orthographic neighbors. Therefore, as the size of orthographic neighbors increases, the ability to keep the processes to store the pseudo characters decreases, perhaps due to the item noise.

In IMF5 and IMF6, which possessed spectral density in the theta- and delta-bands, respectively, stimuli repetition led to a repetition reduction effect on these IMFs. It has been noted that delta activity is related to suppressing irrelevant semantic information (Harmony, 2013). As mentioned before, the interactive activation model would propose that the inputs of visual stimuli activate lexical candidates with similar orthographic or phonological units. Then, the inhibitory connection between words would reduce the activation of each lexical candidate, and only the activity of the target word would accumulate over time. Accordingly, while the same word is presented again, the activity level of lexical candidates is already reduced compared to the first presentation, so the inhibitory mechanism might be omitted. Therefore, stimulus repetition reveals the reduction in theta-delta activities.

It is worth noting that the present study found that P200 activities in IMF4, IMF5, and IMF6 were associated with opposite patterns of repetition effects. Thus, the analyses of IMF4 (alpha band activity) yielded a repetition enhancement effect on P200. Both IMF5 and IMF6 (theta-delta band activity) yielded a repetition reduction effect on P200. Hypothetically, recognizing a target word would involve activating similar words, such as those that are orthographically, phonologically, and semantically similar words. Grainger and Jacobs (1996) proposed the multiple read-out model (MROM), which is based on the interactive activation model, and suggested that word recognition is influenced by local lexical activation of the target word itself and global activity in the mental lexicon induced by partial activation of the target word’s orthographic neighbors. EEG and MEG studies have highlighted the predictive validity of MROM and the interactive activation model for the orthographic neighborhood size effects on early brain activities in around 200 ms after word onsets (Holcomb et al., 2002; Barber et al., 2004; Hsu et al., 2009, 2014). Accordingly, we speculated that two processes might have occurred and influenced the effects of repetition enhancement/reduction. That is, during stimuli repetition, global activation between neighbors would be reduced due to the lateral inhibition between lexical representation, so repeated words would produce less noise as compared with the first presentation. Meanwhile, the local lexical activation of the target word would remain highly activated during repetition as compared with the first presentation.

Admittedly, there were limitations to this study that should be addressed. An important question–which is beyond the scope of this study–is whether the results are confined to the reading of Chinese characters or not. As noted, previous findings of repetition and orthographic neighborhood effects on ERP activities were based on speakers of English, Spanish, and Dutch, which are all more transparent than Chinese in terms of orthographic transparency. Therefore, it seems reasonable to expect that our results would not be confined to languages with opaque orthography such as Chinese. Another limitation to this study is that the EEG measurements were based on the EEG signals to no-go trials, so the present study would not be able to elucidate whether increased EEG activity is associated with improving behavioral performances. Savill and Thierry’s (2012) study indicated that P200 was positively correlated to children’s literacy skills. In a picture naming study by Strijkers et al. (2011), the result showed that the naming latency facilitation (the advantage found for the second presentation over the first presentation) was associated with the repetition enhancement effect on P200. Nevertheless, these findings accentuate the need to explore the nature of repetition enhancement in future work as this topic has received little attention in word recognition studies.

## 5. Conclusion

In conclusion, the present work aims to improve the understanding of repetition effects on EEG data recorded during a visual word recognition task. We suggest that the repetition reduction effect might be the consequence of reduced global lexical activation. The change in alpha-band activity, which indexes lexical access in early stages of word recognition, appears to be associated with the repetition enhancement of P200. This result implies that the neural synchrony hypothesis can also be applied to visual word recognition. In addition to lexical properties, the present study also suggests that the repetition priming effect could be properly deciphered by using an EMD-based technique and clearly demonstrates the potential of EMD-based methods for distinguishing different aspects of repetition priming. Future works on repetition priming in word recognition should consider applying similar methods to separate alpha-band activity and theta-delta band activity.

## Data availability statement

The raw data supporting the conclusions of this article will be made available by the authors, without undue reservation.

## Ethics statement

The studies involving human participants were reviewed and approved by the Human Subject Research Ethics Committee/Institutional Review Board of Academia Sinica, Taiwan. The patients/participants provided their written informed consent to participate in this study.

## Author contributions

C-HH contributed to study conception, experimental design, collecting data, performing the statistical analysis, and writing the manuscript. C-YL contributed to study conception and discussion of the results. Both authors contributed to the article and approved the submitted version.
